# ULK complex organization in autophagy by a C-shaped FIP200 N-terminal domain dimer

**DOI:** 10.1083/jcb.201911047

**Published:** 2020-06-09

**Authors:** Xiaoshan Shi, Adam L. Yokom, Chunxin Wang, Lindsey N. Young, Richard J. Youle, James H. Hurley

**Affiliations:** 1 Department of Molecular and Cell Biology and California Institute for Quantitative Biosciences, University of California, Berkeley, Berkeley, CA; 2 Biochemistry Section, Surgical Neurology Branch, National Institute of Neurological Disorders and Stroke, National Institutes of Health, Bethesda, MD; 3 Molecular Biophysics and Integrated Bioimaging Division, Lawrence Berkeley National Laboratory, Berkeley, CA

## Abstract

The autophagy-initiating human ULK complex consists of the kinase ULK1/2, FIP200, ATG13, and ATG101. Hydrogen-deuterium exchange mass spectrometry was used to map their mutual interactions. The N-terminal 640 residues (NTD) of FIP200 interact with the C-terminal IDR of ATG13. Mutations in these regions abolish their interaction. Negative stain EM and multiangle light scattering showed that FIP200 is a dimer, while a single molecule each of the other subunits is present. The FIP200NTD is flexible in the absence of ATG13, but in its presence adopts the shape of the letter C ∼20 nm across. The ULK1 EAT domain interacts loosely with the NTD dimer, while the ATG13:ATG101 HORMA dimer does not contact the NTD. Cryo-EM of the NTD dimer revealed a structural similarity to the scaffold domain of TBK1, suggesting an evolutionary similarity between the autophagy-initiating TBK1 kinase and the ULK1 kinase complex.

## Teaser

The human ULK complex consists of ULK1/2, FIP200, ATG13, and ATG101. Shi et al. found that the FIP200 N-terminal domain is a C-shaped dimer that binds directly to a single ATG13 molecule and serves as the organizing hub of the complex.

## Introduction

Macroautophagy (henceforward, autophagy) is the conserved eukaryotic cellular process responsible for replenishment of biosynthetic precursors during starvation (Wen and Klionsky, 2016) and engulfment and degradation of molecular aggregates, organelles, intracellular pathogens, and many other cellular substrates (Anding and Baehrecke, 2017; Gomes and Dikic, 2014; Zaffagnini and Martens, 2016). Autophagy proceeds by the de novo formation of a cup-shaped double membrane known as the phagophore or isolation membrane. The phagophore double membrane grows such that it engulfs and isolates its substrates. Upon sealing of the double membrane, the mature structure is referred to as an autophagosome. The autophagosome then fuses with the lysosome, leading to the degradation of the material within the autophagosome. The proteins and protein complexes responsible for these steps have been identified (Mizushima et al., 2011). In mammals (Bento et al., 2016), these include the unc-51–like autophagy activating kinase 1 (ULK1) protein kinase complex, the class III phosphatidylinositol 3-kinase complexes, the phosphatidylinositol 3-phosphate–sensing WIPI proteins, the lipid transporter ATG2 (autophagy related 2), the integral membrane protein ATG9, the ubiquitin-like ATG8 proteins, machinery for conjugating ATG8 protein to lipid membranes, the autophagy adaptors that connect substrates to the ULK1 complex and to ATG8 proteins, and TANK-binding kinase 1 (TBK1), which phosphoregulates autophagy adaptors. The mechanisms by which these protein complexes orchestrate autophagosome initiation, growth, closure, and delivery to the lysosome are being actively sought (Hurley and Young, 2017; Mercer et al., 2018).

The mammalian ULK1 complex is the most upstream of the core protein complexes that make autophagosomes (Itakura and Mizushima, 2010; Karanasios et al., 2013; Karanasios et al., 2016; Koyama-Honda et al., 2013). It is the mammalian counterpart of the yeast Atg1 complex, whose assembly is the main trigger for starvation-induced autophagy in yeast (Kamada et al., 2000). In starvation and TORC1 inhibition, the yeast Atg1 complex assembles from protein kinase Atg1, the bridging subunit Atg13, and the constitutively assembled scaffold Atg17-Atg29-Atg31 to initiate the phagophore (Kamada et al., 2010). The structure of Atg17 has the form of an S-shaped dimer (Ragusa et al., 2012) whose dimensions and curvature are suited to promoting cup-shaped membrane structures (Bahrami et al., 2017). In yeast, there are starvation and TORC1-independent forms of selective autophagy that use Atg1 and Atg13, but with the Atg11 scaffold replacing the Atg17 subcomplex (Yorimitsu and Klionsky, 2005).

The ULK1 complex consists of ULK1 itself, the scaffolding subunit FAK family kinase-interacting protein of 200 kD (FIP200; also known as RB1-inducible coiled-coil protein 1 [RB1CC1]), ATG13, and ATG101 (Ganley et al., 2009; Hosokawa et al., 2009; Jung et al., 2009; Mercer et al., 2009). ULK1 can in most cases be replaced by its paralog ULK2, a closely related serine/threonine kinase that is partially interchangeable within the ULK complex (Mizushima, 2010). ULK1 and ULK2 are the mammalian paralogs of Atg1. ULK1 contains the only known catalytic activity within the complex. The crystal structure of its N-terminal kinase domain is known (Lazarus et al., 2015). The ULK1 kinase targets downstream autophagic machinery including ATG14, VPS34, ATG9, and ATG4 (Papinski and Kraft, 2016; Zachari and Ganley, 2017). ULK1 contains a C-terminal early autophagy targeting/tethering (EAT) domain, which is connected to the kinase domain by an ∼550-residue-long intrinsically disordered region (IDR) and targets ULK1 by binding to a motif in the C terminus of ATG13 (Chan et al., 2009; Hieke et al., 2015).

ATG13 consists of an N-terminal Hop/Rev7/Mad2 (HORMA) domain that dimerizes with the HORMA domain of ATG101 (Qi et al., 2015; Suzuki et al., 2015) and a long C-terminal IDR that binds to FIP200 (Jung et al., 2009; Wallot-Hieke et al., 2018) and ULK1 (Hieke et al., 2015; Wallot-Hieke et al., 2018). The ATG101 HORMA contains an exposed Trp-Phe (WF) finger motif that is important for autophagy (Suzuki et al., 2015). It is not clear what the interaction partner of the WF finger is or how the HORMA dimer fits into the larger organization of the complex.

**Figure 1.**
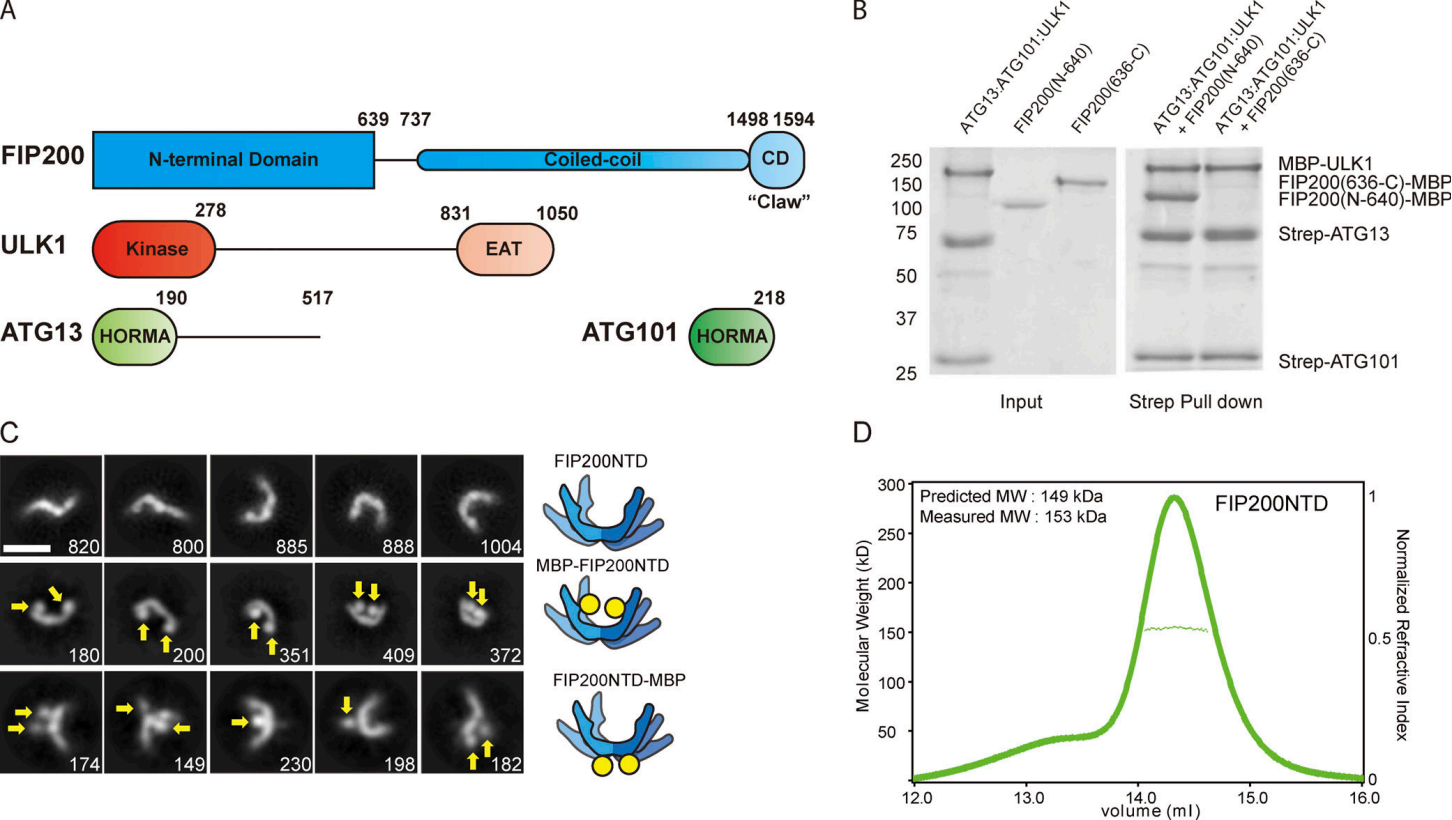
**The FIP200NTD scaffolds the ULK complex as a homodimer.** **(A)** Domain diagram of the ULK complex proteins. **(B)** Pulldown assay of ULK1, ATG13, and ATG101 with FIP200NTD (N-640) and FIP200CTD (636-C). Strep-Tactin resin was loaded with MBP-ULK1:Strep-ATG13:Strep-ATG101 complex to pull down FIP200(N-640)–MBP and FIP200(636-C)–MBP. The pulldown results were visualized by SDS-PAGE and Coomassie blue staining. **(C)** NSEM 2D class averages for FIP200NTD (top), N-terminal MBP–tagged FIP200NTD (middle), and C-terminal MBP-tagged FIP200NTD (bottom). Densities corresponding to MBP tags are labeled with yellow arrows. Scale bar is 20 nm. **(D)** MALS and SEC trace of FIP200NTD shows the predicted and measured molecular weight of the dimeric FIP200NTD. MW, molecular weight.

FIP200 is composed of 1,594 residues, is essential for autophagy, and is considered the functional counterpart of the yeast Atg11 and Atg17 scaffold subunits (Hara et al., 2008; Fig. 1 A). FIP200, however, has no sequence homology to Atg11 and Atg17 apart from the C-terminal 100-residue CLAW domain of FIP200 and Atg11. This is the only portion of FIP200 whose structure is known (Turco et al., 2019). The remainder of FIP200 consists of an ∼640-residue N-terminal domain, followed by an IDR linker and a coiled-coil domain comprising ∼750 residues. Targeting of FIP200 by the autophagy adaptors NDP52 or p62 to mitochondria (Vargas et al., 2019), *Salmonella typhimurium* (Ravenhill et al., 2019), or ubiquitinated cargo (Turco et al., 2019) condensates triggers phagophore initiation, leading to their engulfment. Thus, FIP200 is absolutely central to autophagy initiation. Yet, as one of the largest proteins in the autophagic machinery, FIP200 has been among the most difficult to study in vitro. The lack of reported motifs or sequence homology in the N-terminal 1,500 residues has also slowed progress in understanding this critical part of the autophagy machinery.

In this study, we expressed and purified the human ULK1 complex in order to understand its structural organization. It became clear early in these studies that the full ULK1 complex, with its extensive IDR content and 750-residue FIP200 coiled-coil and dissociable interactions between most of the subunits, is not a typical well-ordered, coassembled constitutive complex. Its dynamic character makes it exceptionally challenging for structural studies. Nevertheless, we were able to use hydrogen-deuterium exchange coupled to mass spectrometry (HDX-MS), EM, and multiangle light scattering (MALS) to map the organization of the complex. These data show that the N-terminal domain (NTD) of FIP200 serves a C-shaped dimeric hub for assembly of the ULK1 complex.

## Results

### FIP200NTD assembles with ULK1, ATG13, and ATG101

Given that FIP200 is essential for autophagy and is the largest protein in the ULK1 complex, we began with the hypothesis that some part of FIP200 was likely to be the main hub organizing the complex. We sought to identify the minimal domain needed for the assembly of the core ULK1 complex. Both the NTD (1–640) and the C-terminal domain (CTD; 636–1594; Fig. 1 A) of FIP200 were expressed in HEK 293T cells as N-terminal GST and C-terminal MBP fusions. The remaining three subunits, ULK1, ATG13, and ATG101, were separately coexpressed with each other and purified. Purified FIP200NTD–MBP and FIP200 CTD–MBP were used in a pulldown assay with the ternary ULK1:ATG13:ATG101 complex. These experiments showed there was an interaction between ULK1:ATG13:ATG101 and the FIP200NTD, but not the CTD (Fig. 1 B).

### The FIP200NTD is a C-shaped dimer

**Figure S1.**
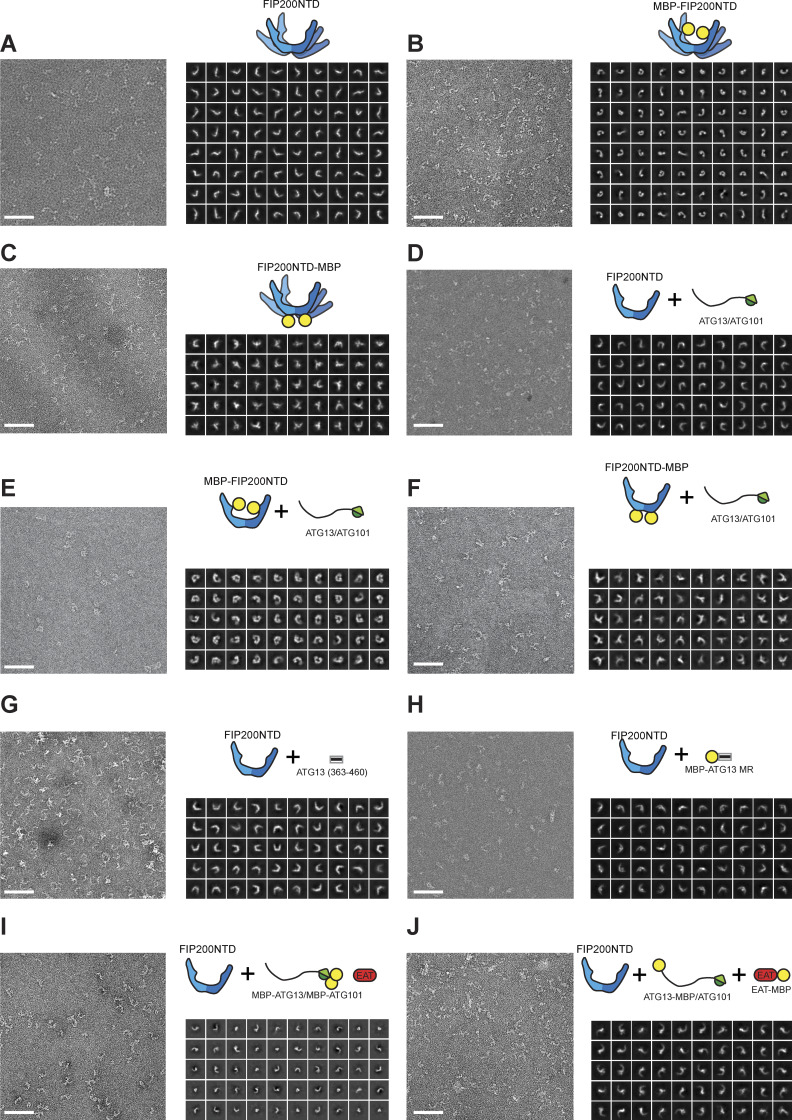
**NSEM of FIP200NTD complexes.** **(A–J)** Representative micrograph, schematic, and gallery of 2D class averages for each negative stain dataset. Scale bar on each micrograph is 80 nm.

We characterized the overall size and shape of FIP200NTD using negative stain EM (NSEM) and multiangle light scattering (MALS). NSEM 2D classification of FIP200NTD showed a variety of shapes (Fig. 1 C; Fig. S1, A–C; and Table S1), with maximum dimensions ranging from 10 to 24 nm. Many of the 2D averages were in the shape of the letter C. Others resembled singly bent rods or S shapes (Fig. 1 C). MBP tags were fused to either the N or C terminus in order to mark the location of each end by the presence of additional density compared with untagged FIP200NTD. 2D class averages of the MBP N-terminal–tagged construct displayed a similar variety of shapes, with two additional densities corresponding to two MBP tags at the tips of the density (Fig. 1 C, see arrows). This observation suggested that FIP200NTD is a dimer and that the N termini are distal to the dimer interface. The C-terminal MBP tags were localized near the center of the C shape. The C-terminal tags displayed more dispersed positions, with either one or two tags visualized. Together, the MBP tags suggest the FIP200NTD forms a dimeric structure with the C termini close to the dimer interface and the N termini at the tips.

To assess the oligomeric state of FIP200 by an independent technique, we used size exclusion chromatography (SEC) coupled to MALS to analyze the molecular weight of FIP200NTD in solution. The SEC-MALS resulted in a single peak with a molecular weight of 153 kD (Fig. 1 D). This value corresponds closely to the predicted molecular weight of 149 kD of the FIP200NTD dimer.

### Mapping FIP200NTD interactions with the rest of the ULK1 complex

**Figure 2.**
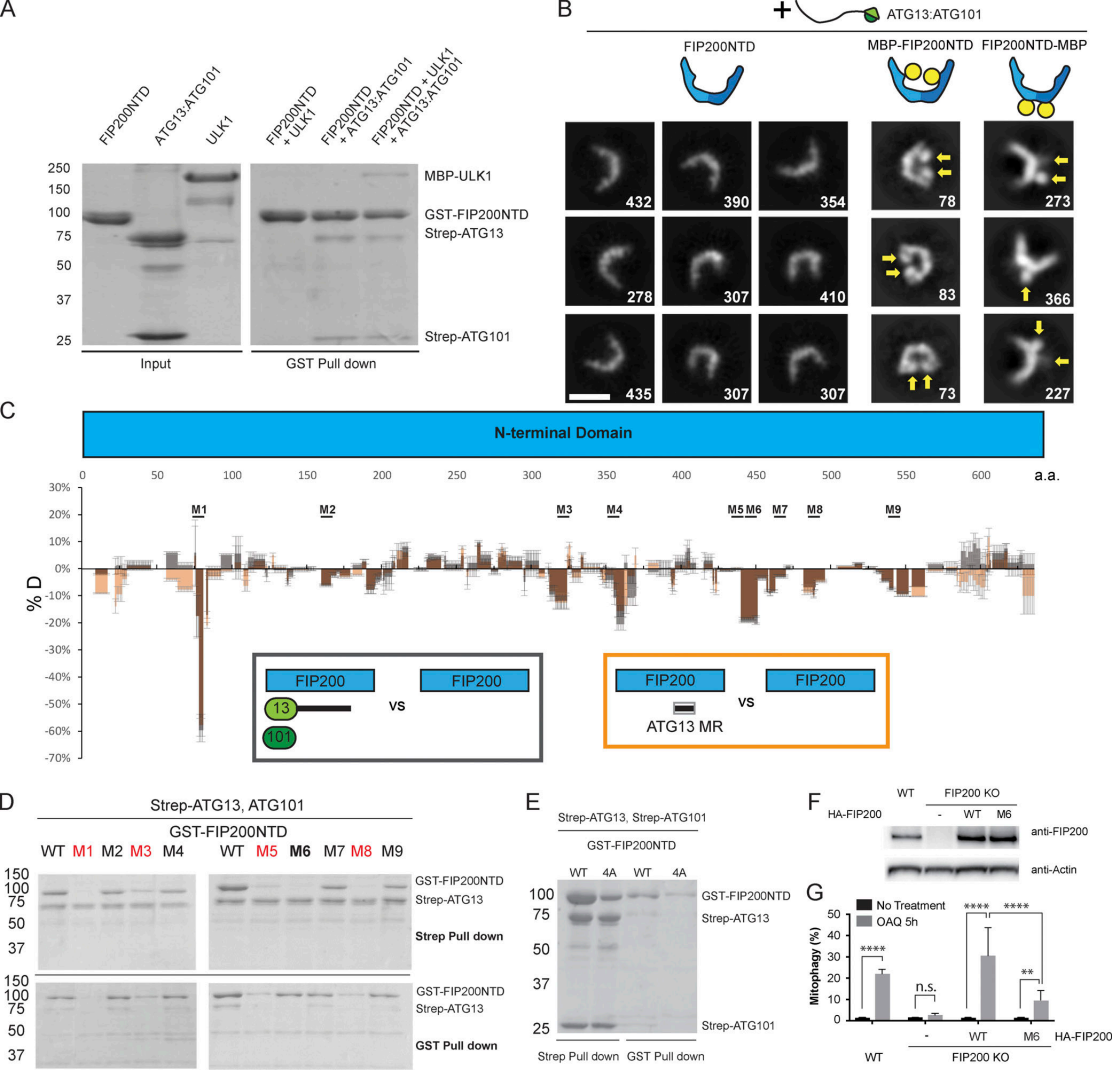
**Mapping the FIP200NTD interaction sites with ATG13 and ATG101.** **(A)** Pulldown assay of FIP200NTD with ULK1, ATG13:ATG101, and both. GSH resin was loaded with GST-FIP200NTD to pull down MBP-ULK1, Strep-ATG13:Strep-ATG101, and both. The pulldown results were visualized by SDS-PAGE and Coomassie blue staining. **(B)** NSEM 2D class averages of FIP200NTD:ATG13:ATG101, MBP-FIP200NTD:ATG13:ATG101, and FIP200NTD–MBP:ATG13:ATG101. Densities corresponding to MBP tags are labeled with yellow arrows. Scale bar is 20 nm. **(C)** Difference of HDX percentages of the FIP200NTD alone versus FIP200NTD:ATG13:ATG101 (black) or FIP200NTD:ATG13MR (orange) at 6-s time point. Brown represents the overlay of black and orange. Sites of mutation are labeled above matching residues. All values are mean ± SD. **(D)** Pulldown assays of mutant FIP200NTD constructs (M1–M9) and WT with ATG13:ATG101. Both GSH and Strep-Tactin resin were used to pull down GST-FIP200NTD:Strep-ATG13:ATG101 complex from lysate of overexpressing HEK cells. The pulldown results were visualized by SDS-PAGE and Coomassie blue staining. Mutants marked red are the mutants that had a loss of FIP200NTD stability; mutants marked black are the mutants that had no evident loss of FIP200NTD stability or any effect on ATG13:ATG101 pulldown; Mutant marked bold are the mutant that had no evident loss of FIP200NTD stability but eliminated the interaction with ATG13:ATG101. **(E)** Pulldown assay of mutant FIP200NTD constructs (4A) and WT with ATG13:ATG101. **(F)** Expression level of FIP200 in samples used for mitophagy analysis. **(G)** Quantification of mito-mKeima ratiometric FACS analysis of WT or FIP200 KO cells reexpressing WT or mutant FIP200 after 5 h of OAQ treatment. *n* = 3 biological replicates. All values are mean ± SD. P values: **, < 0.01; ****, < 0.0001. n.s., not significant; OAQ, Oligomycin, Antimycin A, and Quinoline-Val-Asp-Difluorophenoxymethyl Ketone (QVD). a.a., amino acid.

We sought to determine the minimal region(s) of ULK1:ATG13:ATG101 interacting with FIP200NTD. Strep-ATG13:Strep-ATG101 was efficiently pulled down by FIP200NTD alone, but MBP-ULK1 was not (Fig. 2 A). Pulldown of ULK1 was recovered in the presence of ATG13:ATG101 (Fig. 2 A). This shows that ATG13:ATG101 interacts directly with FIP200NTD, while ULK1 recruitment to FIP200NTD depends on the presence of ATG13:ATG101. We characterized the minimal FIP200NTD:ATG13:ATG101 subcomplex by NSEM. 2D classification showed distinct C shapes, as seen for FIP200NTD alone (Fig. 2 B; Fig. S1, D–F; and Table S1). However, we did not observe any of the bent rods and S shapes seen in the absence of ATG13:ATG101. Thus, ATG13:ATG101 stabilizes the C-shaped conformation of FIP200NTD. No extra density corresponding to HORMA dimers was seen in the 2D averages, suggesting that the position of the HORMA dimer is not ordered with respect to the FIP200NTD.

**Figure S2.**
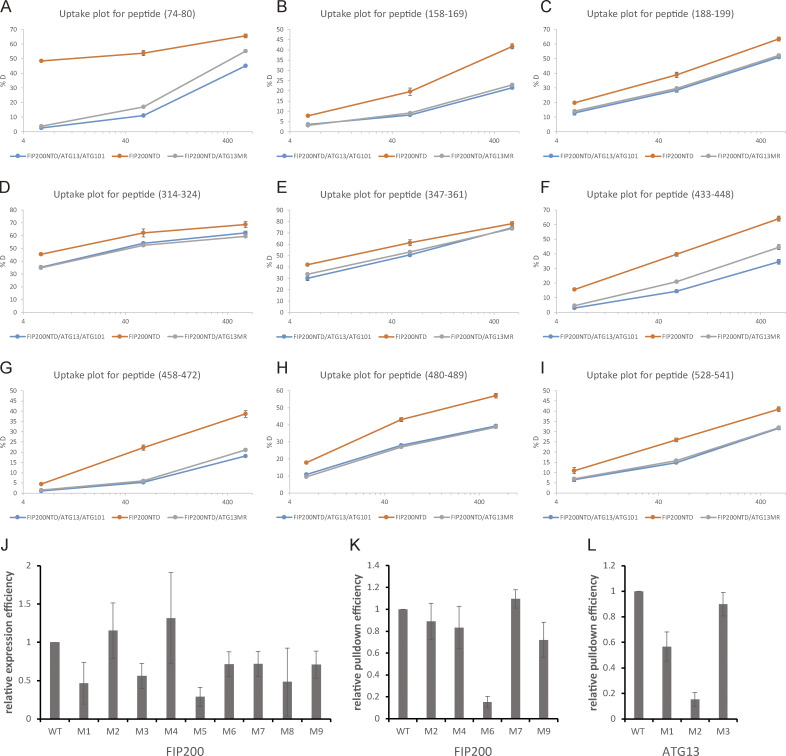
**Deuterium uptake plot for peptides in FIP200NTD with significant differences and quantification of pulldown assay.** **(A)** Deuterium uptake plot for peptide (74–80). **(B)** Deuterium uptake plot for peptide (158–169). **(C)** Deuterium uptake plot for peptide (188–199). **(D)** Deuterium uptake plot for peptide (314–324). **(E)** Deuterium uptake plot for peptide (347–361). **(F)** Deuterium uptake plot for peptide (433–448). **(G)** Deuterium uptake plot for peptide (458–472). **(H)** Deuterium uptake plot for peptide (480–489). **(I)** Deuterium uptake plot for peptide (528–541). **(J)** Quantification of relative expression efficiency for GST-FIP200NTD in Fig. 2 D. **(K)** Quantification of relative pulldown efficiency for GST-FIP200NTD in Fig. 2 D. **(L)** Quantification of relative pulldown efficiency for GST-ATG13MR in Fig. 3 D. All values are mean ± SD.

We used HDX-MS to systematically compare FIP200NTD:ATG13:ATG101 with FIP200NTD alone to identify regions in FIP200NTD interacting with ATG13:ATG101 (Fig. 2 C; Fig. S2, A–I; and Data S1). In general, FIP200NTD had lower HDX when bound to ATG13:ATG101, as the global HDX difference with FIP200NTD alone was negative (−3.6% for all peptides combined), consistent with the overall stabilization seen in NSEM 2D class averages. Significant protection (between −10% and −100%) was seen in nine regions of the FIP200NTD. We mutated side chains within each region and tested the effects on ATG13:ATG101 binding. Each region was converted a poly Gly-Ser sequence of equal length to the WT region being replaced. GST and Strep pulldown assays showed that mutation of regions 1 (73–80), 3 (319–326), 5 (435–442), and 8 (482–489) impaired the stability of FIP200NTD (Fig. 2 D, red, see bottom gel; and Fig. S2 J), and no conclusion could be drawn as to whether their interaction with ATG13-:ATG101 was direct or not. Mutants M2 (158–165), M4 (350–357), M7 (464–471), and M9 (537–544) had no evident loss of FIP200NTD stability or any effect on ATG13:ATG101 pulldown (Fig. 2 D, black, top gel; and Fig. S2, J and K). Thus, ATG13:ATG101 binding leads to a large overall decrease in FIP200NTD dynamics extending across regions beyond those essential for ATG13:ATG101 binding. Mutation of region M6 (443–450) had no loss of protein expression while eliminating the interaction with ATG13:ATG101 (Fig. 2 D, bold; and Fig. S2, J and K). Region 6 is therefore a major locus of ATG13:ATG101 binding.

The 582–585 region that was previously proposed to be the interaction site for ATG13 (Chen et al., 2016) had a slight decrease in protection, inconsistent with the expectation that the direct interacting regions should show substantial increases in protection. We replicated the 582–585 4A mutant from that study (Chen et al., 2016). ATG13:ATG101 pulled down less FIP200NTD (4A) than WT (Fig. 2 E), but we attribute this to FIP200NTD (4A) being expressed at a much lower level than WT FIP200NTD. Taking the observation that the 4A mutant reduces FIP200NTD stability together with the lack of an increase in HDX protection in this region upon ATG13 binding, we ascribe the decrease in binding and the phenotype observed by Chen et al. (2016) to decreased stability of the FIP200NTD dimer, rather than to a direct interaction.

**Figure S3.**
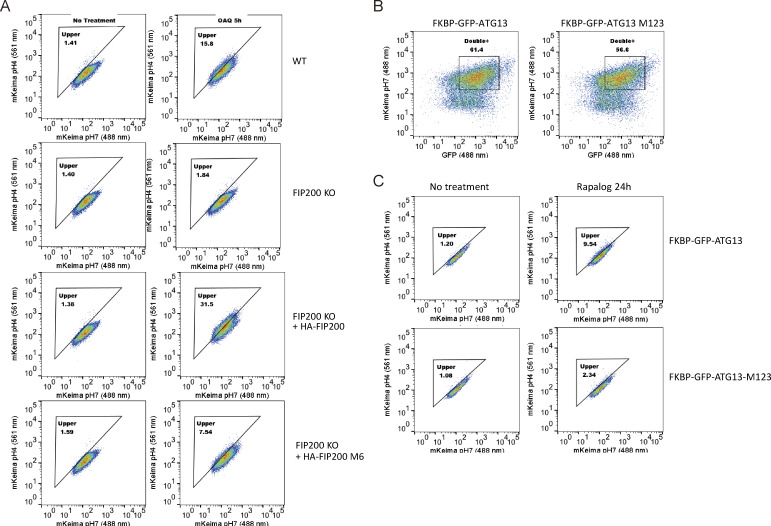
**FACS analysis of the FIP200-ATG13 interaction in mitophagy.** **(A)** FACS plots showing mito-mKeima ratio (561/488 nm) for Fig. 2 G. **(B)** The WT ATG13 and ATG13 M123 are expressed at similar levels in cells used in Fig. 3 E. **(C)** FACS plots showing mito-mKeima ratio (561/488 nm) for Fig. 3 E. OAQ, Oligomycin, Antimycin A, and QVD.

Having mapped the interaction sites for ATG13:ATG101 on FIP200, we sought to investigate the function of these sites in an autophagic process. We assessed mitophagy in HeLa cells using a mito-mKeima assay (Vargas et al., 2019). The M6 mutant construct that disrupted the FIP200-binding site for ATG13 interface was transfected in HeLa cells to determine its effects (Fig. 2, F and G). FIP200 knockout (KO) HeLa cells showed a severe defect in mitophagy relative to the WT HeLa cells (Fig. 2 G). Transient transfection of WT FIP200 and FIP200 M6 corresponding to region 6 (443–450 mutated to GSSGGSSG) was performed as described earlier (Vargas et al., 2019), and mitophagy was assayed. After 5-h Oligomycin, Antimycin A, and QVD treatment to induce mitophagy, cells were analyzed using FACS (Fig. S3 A). The FIP200 M6 mutant exhibited a fivefold reduction in mitophagy compared with WT FIP200 in the KO rescue experiment, confirming that FIP200 binding to ATG13 is important for its function.

### Mapping the FIP200-binding site on ATG13

**Figure 3.**
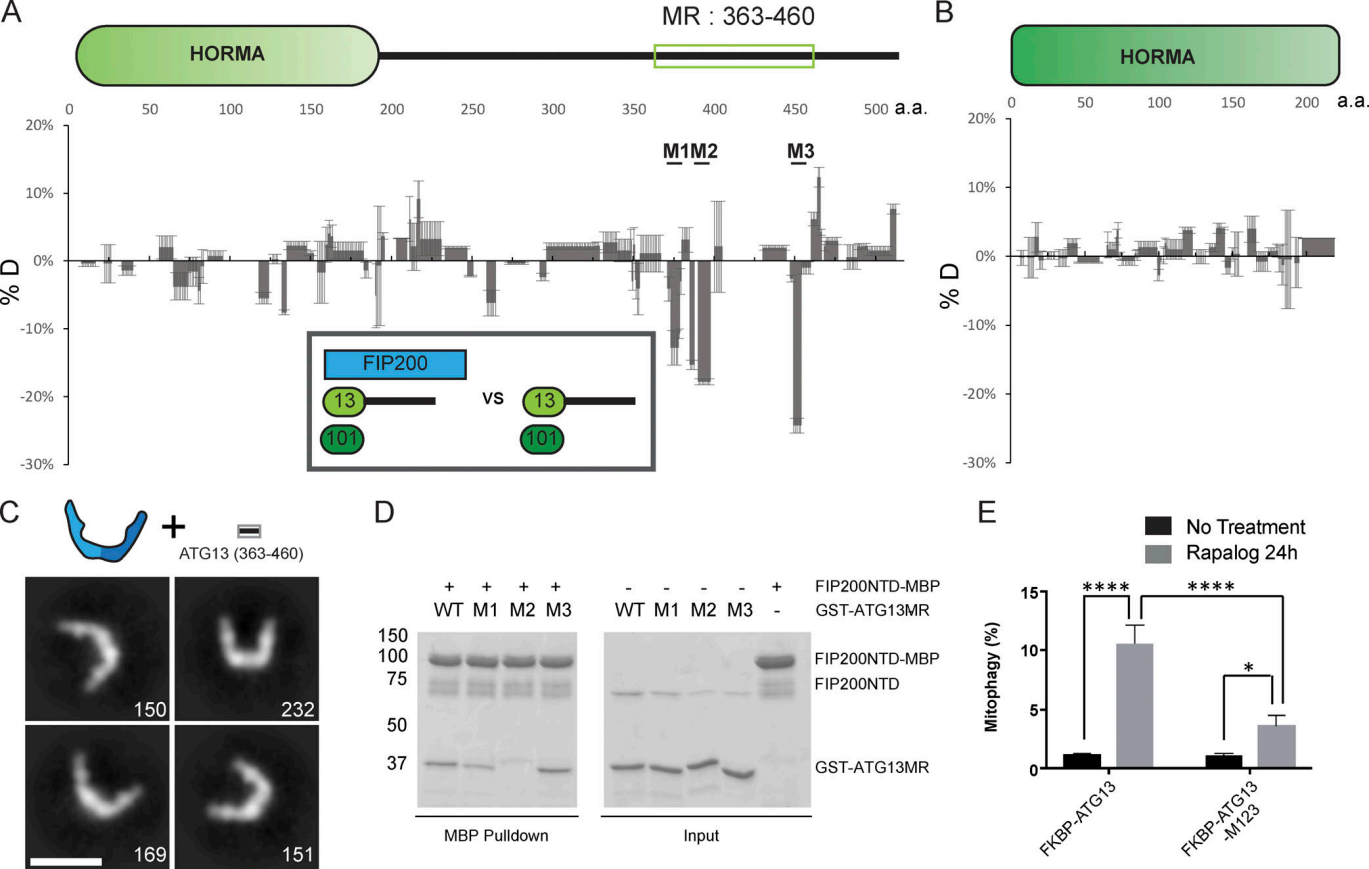
**Mapping the FIP200NTD binding sites on ATG13.** **(A)** Difference of HDX percentages of the ATG13 in ATG13:ATG101 versus ATG13:ATG101:FIP200 at 6-s time point. Sites of mutation are labeled above matching residues. The MR region is highlighted with the unfilled rectangular green box, as labeled. All values are mean ± SD. **(B)** Difference of HDX percentages of the ATG101 in ATG13:ATG101 versus ATG13:ATG101:FIP200. All values are mean ± SD. **(C)** NSEM 2D class averages of FIP200NTD:ATG13 MR complex. The two color shades in the cartoon C-shape denote the two FIP200NTD monomers in the dimer. Scale bar is 20 nm. **(D)** Pulldown assays of mutant ATG13MR constructs (M1–M3) and WT with FIP200NTD. Amylose resin was used to pull down purified GST-ATG13MR:FIP200NTD-MBP complex. The pulldown results were visualized by SDS-PAGE and Coomassie blue staining. **(E)** Quantification of mito-mKeima ratiometric FACS analysis of HeLa cells stably expressing mito-mKeima–P2A-FRB-Fis1 and FKBP-GFP-ATG13 or mutant after 24 h of Rapalog treatment. *n* = 3 biological replicates. All values are mean ± SD. P values: *, = < 0.05; ****, < 0.0001. n.s., not significant. a.a., amino acid.

We used HDX-MS to compare FIP200NTD:ATG13:ATG101 with ATG13:ATG101 and to identify the regions of ATG13:ATG101 involved in assembly with FIP200NTD. In general, the ATG13 middle region (ATG13MR; 363–460) showed reduced HDX (−6.5% for all peptides combined) when bound to the FIP200NTD dimer (Fig. 3 A and Data S1). Both the HORMA domain of ATG13 and of ATG101 only showed slight differences, below the 10% threshold of significance (Fig. 3 A and Fig. 3 B). These data suggest that the ATG13 MR, not the HORMA dimer, is the FIP200NTD binding site. This is consistent with the finding that deletion of ATG13 isoform2 348–373 blocks FIP200 interaction (Wallot-Hieke et al., 2018). This is also consistent with the NSEM result that in the FIP200NTD:ATG13:ATG101, the HORMA domain dimer density was averaged out in 2D classifications (Fig. 2 B).

To compare the properties of the isolated ATG13MR with the full ATG13:ATG101 subcomplex, we compared the HDX of FIP200NTD:ATG13 MR with FIP200NTD alone (Fig. 2 C; Fig. S2, A–I; and Data S1). The presence of the ATG13MR led to a pattern of differences identical to those induced by full-length ATG13:ATG101 (Fig. 2 C). Furthermore, NSEM analysis of the FIP200NTD:ATG13MR sample showed stable C shapes like those of the FIP200NTD:ATG13:ATG101 (Fig. 2 B, Fig. 3 C, Fig. S1 G, and Table S1). Thus, the 98-residue ATG13MR fully recapitulates the properties of the full ATG13:ATG101 subcomplex with respect to its ability to bind and rigidify FIP200. Three regions in the ATG13MR (371–378, 390–397, and 446–453) were selected and mutated to poly Gly-Ser sequences of equal length to the WT region being replaced. GST-tagged ATG13MR constructs and FIP200NTD–MBP were purified and used in MBP pulldown assays (Fig. 3 D and Fig. S2 L). Mutation of region 2 (390–397) largely impaired the interaction between ATG13MR and FIP200NTD, while mutation of region 1 (371–378) moderately impaired the interaction. A slight effect was observed when using mutation of region 3 (446–453).

It was previously found that in *atg13* KO mouse embryonic fibroblasts rescued by ATG13 construct expression, loss of the N-terminal part of the ATG13MR had no effect on starvation-induced autophagy and only a slight effect on torin-induced autophagy (Wallot-Hieke et al., 2018). We assessed mitophagy in HeLa cells using a mito-mKeima assay (Vargas et al., 2019). To completely disrupt the FIP200 binding, all three regions in ATG13MR (371–378, 390–397, and 446–453) were mutated to poly Gly-Ser sequences of equal length to the WT region being replaced to generate the M123 mutant. The WT and M123 mutant were then stably expressed at a similar level in HeLa cells, and mitophagy was assayed using a previously described chemical-induced dimerization (CID) system (Vargas et al., 2019; Fig. 3 E; and Fig. S3, B and C). Compared with ATG13 WT, ATG13 M123 mutant triggered a significantly reduced mitophagy response (threefold reduction). This contrasts with a report that mutating a subset of the FIP200-binding region of ATG13, residues 348–373, does not impact starvation-induced autophagy in MEFs (Wallot-Hieke et al., 2018). These data show that the three FIP200-binding regions of ATG13 analyzed here are functionally important in mitophagy.

### ULK1-EAT interactions with FIP200NTD:ATG13:ATG101

**Figure 4.**
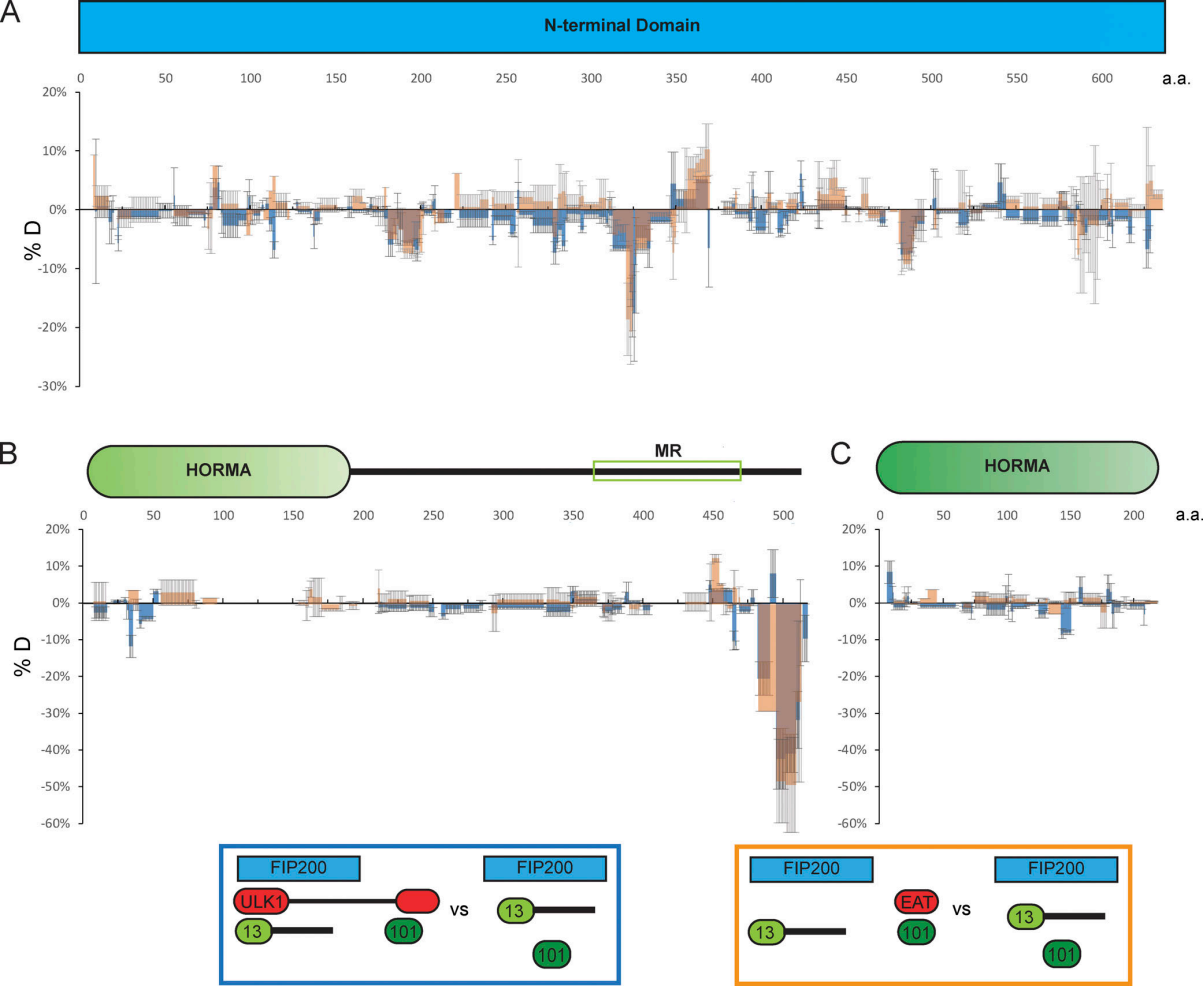
**Interactions between ULK1 and the FIP200:ATG13:ATG101 subcomplex.** **(A–C)** Difference of HDX percentages of the FIP200NTD (A), ATG13 (B), and ATG101 (C) in ATG13:ATG101:FIP200NTD versus in ATG13:ATG101:FIP200NTD:ULK1(blue) or ATG13:ATG101:FIP200NTD:ULK1 EAT(orange) at the 60-s time point. In B, the ATG13MR is highlighted by the unfilled green rectangle. Brown represents the overlay of blue and orange. All values are mean ± SD. a.a., amino acid.

**Figure S4.**
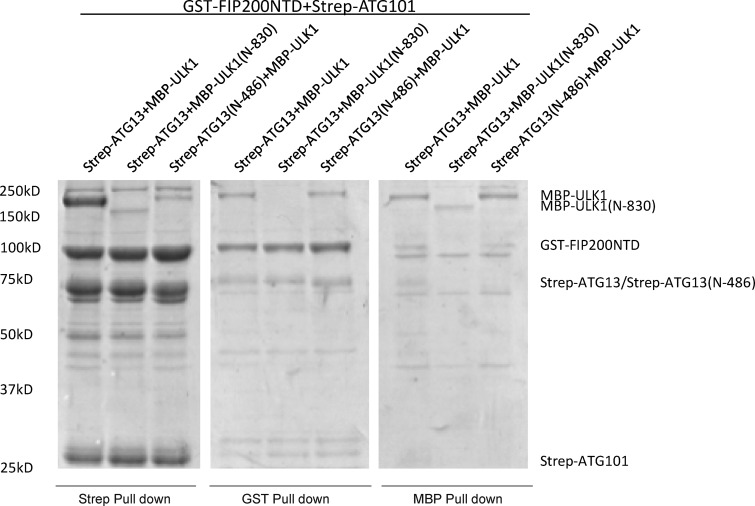
**Pulldown assays of the ULK1 complex with FIP200NTD, ATG13, and ULK1 constructs.** GSH, Strep-Tactin, and Amylose resin were used to pull down GST-FIP200NTD:Strep-ATG13:Strep-ATG101:MBP-ULK1 complex from lysate of overexpressing HEK cells. The pulldown results were visualized by SDS-PAGE and Coomassie blue staining.

The FIP200NTD:ATG13:ATG101:ULK1 complex was purified from HEK 293 cells and subjected to HDX-MS analysis to map the ULK1 binding sites on the rest of the complex. FIP200NTD peptide 319–326 and ATG13 peptide 482–517 showed significantly decreased HDX in the presence of ULK1 (Fig. 4, A and B). In contrast, ATG101 had no significant HDX changes upon the addition of ULK1 (Fig. 4 C). The HDX changes in FIP200 and ATG13 peptides suggested that these two regions could serve as the ULK1 binding site. FIP200 region 319–326 is also important for FIP200NTD stability (M3; Fig. 2 D and Fig. S2 J). It was reported that the EAT domain of ULK1 interacts with the C terminus of ATG13 (Wallot-Hieke et al., 2018), as anticipated from the homology between the corresponding regions of yeast Atg1 and Atg13 (Fujioka et al., 2014; Stjepanovic et al., 2014). Consistent with this, ULK1 EAT alone induced essentially identical HDX changes in both FIP200NTD and ATG13 compared with full-length ULK1 (Fig. 4, A and B). To investigate whether ULK1, FIP200NTD, and ATG13 form a three-way interface, ULK1(N-830/ΔEAT) and ATG13(N-486/ΔC) were assayed by pulldown in the presence of all four ULK1 complex subunits. GST–FIP200NTD can still pull down some MBP-ULK1 or vice versa even in the presence of ATG13-ΔC (Fig. S4). This demonstrates that once ATG13 brings FIP200 and ULK1 together, a direct interaction exists between FIP200NTD and ULK1 EAT.

### An asymmetric ULK1 complex with 1:2:1:1 ULK1:FIP200:ATG13:ATG101 stoichiometry

**Figure 5.**
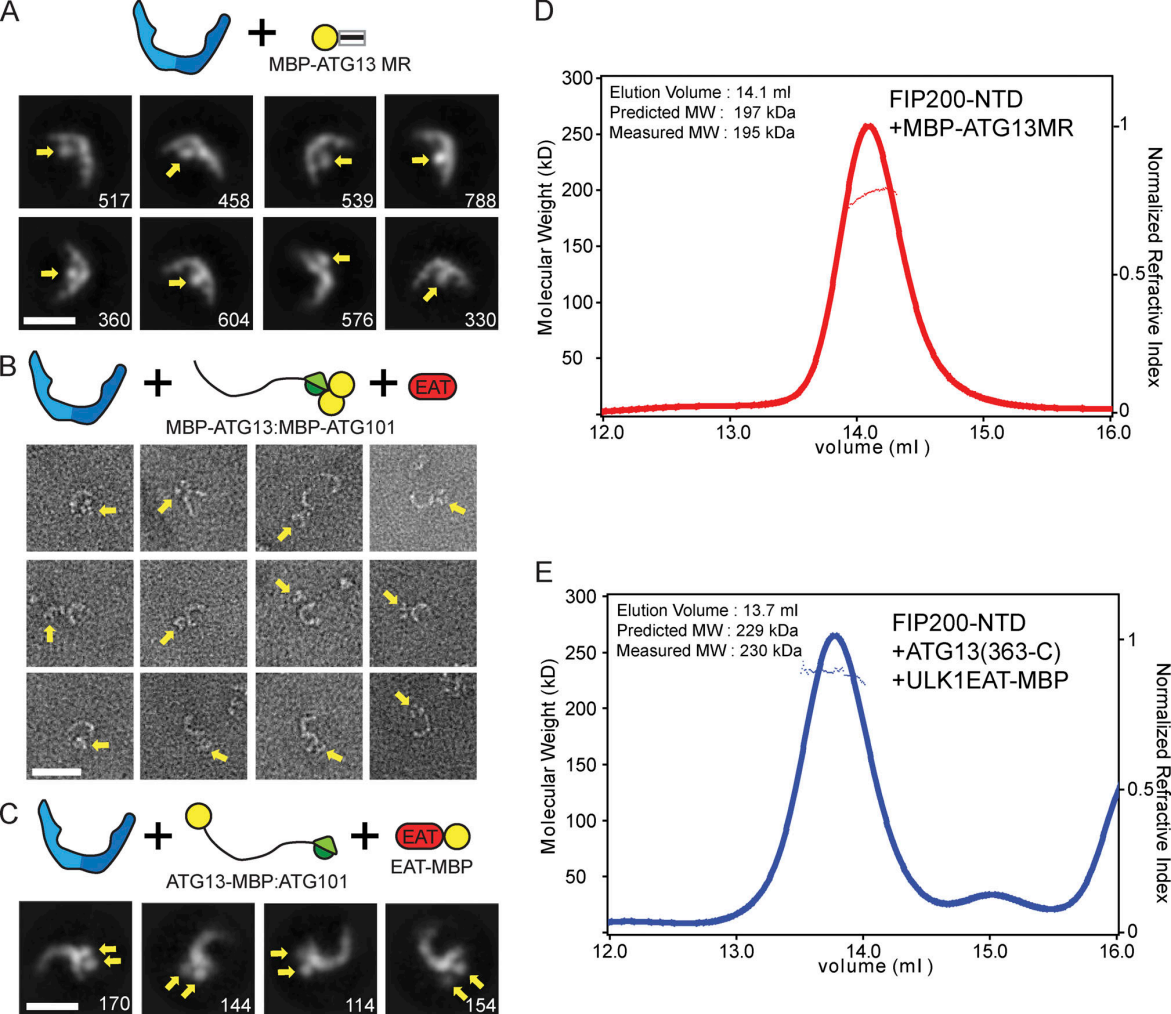
**The ULK complex has unequal subunit stoichiometry.** **(A)** Negative stain 2D class averages of FIP200NTD:MBP-ATG13MR complex. Densities corresponding to MBP tags are labeled with yellow arrows. Scale bar is 20 nm. **(B)** Single particles of FIP200NTD:MBP-ATG13:MBP-ATG101:ULK1 EAT complex. Density for dual MBP-tagged HORMA domains is highlighted with yellow arrows. Scale bar is 20 nm. **(C)** 2D class averages of FIP200NTD:ATG13-MBP:ATG101:ULK1 EAT–MBP complex. Densities corresponding to MBP tags are labeled with yellow arrows. Scale bar is 20 nm. **(D and E)** MALS traces for FIP200NTD:MBP-ATG13MR (D) and FIP200NTD:ATG13(363-C):ULK1 EAT–MBP (E). MW, molecular weight.

Having defined the minimal interacting regions responsible for assembly of the ULK1 complex, we sought to localize them in space relative to the FIP200NTD scaffold and to understand their stoichiometry. First, FIP200NTD:MBP–ATG13MR was analyzed by NSEM. 2D class averages showed that only one MBP density could be seen near the center of “C,” suggesting that only one molecule of ATG13 binds per FIP200NTD dimer (Fig. 5 A, Fig. S1 H, and Table S1). This suggests that the ATG13 binding site spans both FIP200NTD monomers in the C-shaped dimer.

To determine if the ATG13:ATG101 HORMA dimer was structurally ordered with respect to the FIP200NTD scaffold, we fused MBP tags to each protein. This more than doubled the effective mass of these domains, making them visible by NSEM. In single particles of FIP200NTD:MBP-ATG13:MBP-ATG101:ULK1 EAT, a trimer density can be seen in the vicinity of the FIP200NTD dimer, corresponding to density for the HORMA dimer and the two MBP tags present (Fig. 5 B, Fig. S1 I, and Table S1). 2D classification of the complex showed a stable FIP200NTD dimer, but most averages had no additional densities for the MBP tags (Fig. S1 I). We interpret this to mean that the HORMA dimer does not directly interact with, and is not ordered with respect to, FIP200NTD.

We next performed NSEM with ATG13-MBP and ULK1 EAT–MBP. MBP tags placed at the ATG13 C terminus and on the ULK1 EAT domain were colocalized as seen by the presence of two extra density lobes present at the tip of one arm of the FIP200NTD dimer (Fig. 5 C, Fig. S1 J, and Table S1). 2D classification showed that the ULK1 EAT and the C terminus of ATG13 are located near one of the tips of the FIP200NTD C shape.

The results of the MBP tagging experiments implied that the ULK1 complex is asymmetric and has nonequal subunit stoichiometry. To test this, we used MALS to determine the stoichiometry of the ULK1 complex by direct determination of the molecular mass. FIP200NTD:MBP–ATG13MR and FIP200NTD:ATG13(363-C):ULK1 EAT–MBP both showed molecular weights consistent with a stoichiometry of two molecules of FIP200NTD for each one molecule of all other components (Fig. 5, D and E). The measured molecular weights of 195 kD and 230 kD correspond to the expected mass of complexes with 2:1 ratios between the FIP200NTD and the other subunits (197 kD, and 229 kD, respectively). Taken together, the NSEM and MALS data show that the FIP200NTD dimer assembles asymmetrically with one copy each of ATG13, ATG101, and ULK1.

### Cryo-EM structure of ATG13MR–bound FIP200NTD

**Figure S5.**
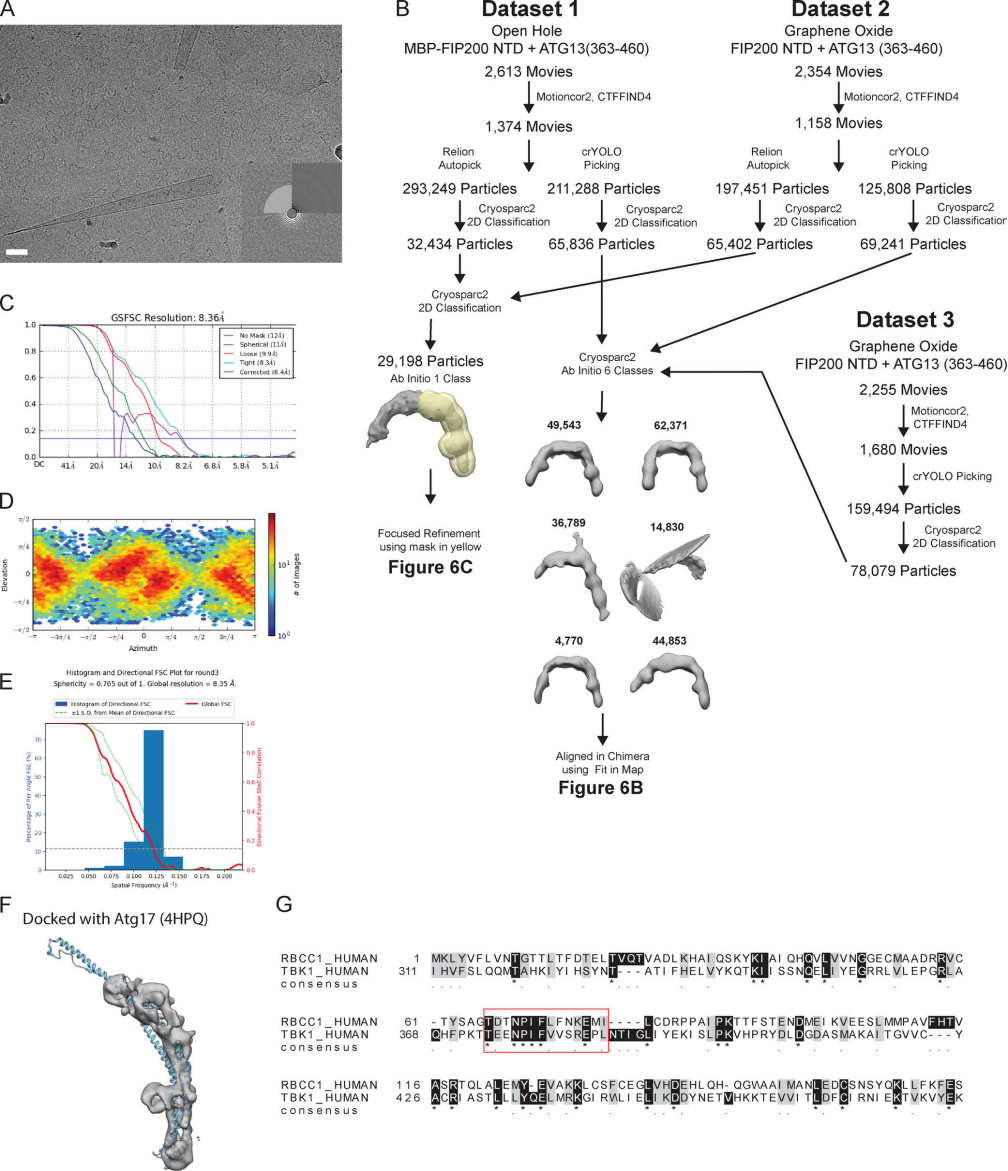
**Cryo EM workflow.** **(A)** Representative micrograph from Dataset 2. Scale bar is 40 nm. **(B)** Data processing scheme for final cryo-EM reconstructions. **(C)** FSC curves of final cryo-EM reconstruction of FIP200NTD. **(D)** Orientation parameters of final particle alignments. **(E)** 3DFSC plot of the final map. **(F)** Fit of Atg17 structure (PDB accession no. 4HPQ, blue) into our cryo-EM density. **(G)** Sequence alignment of FIP200 (RB1CC1) and human TBK1. The red box indicates the region with the highest conservation and that contains the NPIF motif.

**Figure 6.**
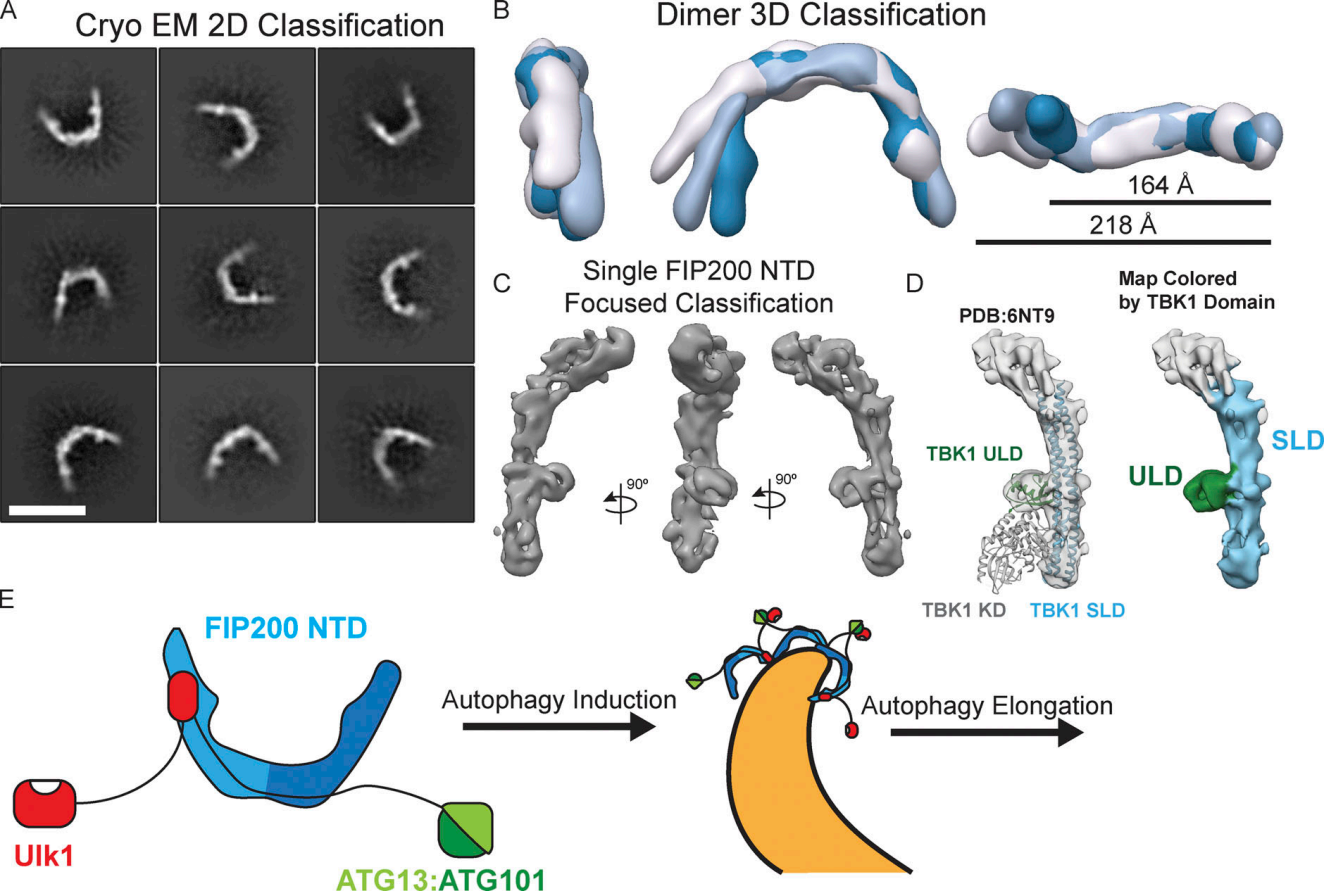
**Cryo-EM reconstruction of the FIP200NTD:ATG13MR complex and model for ULK complex assembly.** **(A)** Cryo-EM 2D class averages of FIP200NTD:ATG13MR. Scale bar is 20 nm. **(B)** 3D reconstructions of dimeric FIP200NTD overlaid with measurements from tip-to-tip ranging from 164 Å to 218 Å across. The different colors correspond to different density maps from different 3D classes. **(C)** Views of masked final 3D reconstruction of FIP200NTD monomer. **(D)** Fit of TBK1 structure into the final map (left) and colored by ULD (green) and SLD (right; blue). **(E)** Model for asymmetric FIP200 ULK complex assembly and proposed formation of higher oligomers during autophagy initiation.

We used cryo-EM to investigate the architecture of the FIP200NTD dimer:ATG13 MR complex at higher resolution. The narrow extended C shape and flexible nature of FIP200 made it a challenging sample for both sample preparation and data collection. Nevertheless, the use of several technical improvements made it possible to obtain an intermediate resolution structure. Graphene oxide was used to protect the FIP200NTD dimer from the air–water interface and allowed collection of high-contrast micrographs (Fig. S5 A). Accurate particle picking was critical for the centering of the FIP200NTD C shape. The neural network–based crYOLO picker (Wagner et al., 2019) was trained by manual picking and used to autopick micrographs from three datasets (Fig. S5 B and Table S2). 2D classification showed the FIP200NTD dimer in an array of more or less open conformational states similar to that seen in the NSEM data (Fig. 6 A). A distinct bump was observed on the inner rim of the dimer. After 3D classification, full dimer maps were resolved between 12 Å and 15 Å resolution (Fig. 6 B and Fig. S5 B). When overlaid, these maps show a large range of tip-to-tip distances, spanning 160 Å to 220 Å across.

Focused refinement with a mask around a single FIP200NTD was performed and yielded a final resolution of 9 Å (Fig. 6 C; and Fig. S5, B–E). At this resolution, helical densities can be seen that run along the length of the C shape. Additionally, more features are seen at density within the dimer C shape.

To interpret this moderate resolution map, given the lack of preexisting atomic structures for FIP200 outside of the CLAW domain, we relied on structure prediction servers. Robetta analysis (Song et al., 2013) suggested the TBK1 scaffold-like domain (SLD) and ubiquitin-like domain (ULD; Shu et al., 2013) as the basis for a structural model. Docking of TBK1 ULD and SLD (Shu et al., 2013; Zhang et al., 2019) showed a good fit into our density (Fig. 6 D, left), while another candidate, the yeast Atg17 structure, did not (Fig. S5 F). These domains from TBK1 are composed of 429 residues that would not account for the FIP200NTD construct (1–640; Fig. 6 D, right). The dimer interface of the FIP200NTD dimer remains unmodeled at our current resolution. The bump seen in 2D averages corresponds to the ULD, and the backbone of the arm forms a helical bundle that is similar in spacing and structure to the SLD of TBK1 (Fig. 6 D). TBK1 has a conserved interface positioned between the ULD domain and the scaffolding domain containing residues NPIF (376–380; Shu et al., 2013; Zhang et al., 2019). FIP200 contains the same sequence near the end of the predicted ULD in residues 67–72 (Fig. S5 G). This region corresponds with a region of conserved primary sequence between FIP200 and TBK1.

## Discussion

The ULK1 complex has a central role in autophagy initiation, yet its structural organization (Lin and Hurley, 2016) is not nearly as well understood as that of the other major complexes of autophagy initiation, class III phosphatidylinositol 3-kinase complexes–C1 and ATG16L1-ATG5-ATG12. In particular, there have been no structural data for the largest subunit, FIP200, apart from very recent structures of the Claw domain (Turco et al., 2019), representing <10% of the total mass of the protein. Here, we substantially filled these gaps at two levels. We mapped the overall structural organization and showed that the FIP200NTD is the hub about which the larger complex is organized. We infer that the FIP200 CC and Claw, the ATG13:ATG101 HORMA dimer, and the ULK1 kinase domain probably project away from the hub, given that they are nonessential for complex formation, do not show HDX protection patterns consistent with interactions, or both (Fig. 6 E). These three projecting regions appear not to interact with the hub at all under the conditions of these studies.

FIP200 is often considered the functional orthologue of Atg17 in mammals (Lin and Hurley, 2016; Mizushima, 2010). Like Atg17, FIP200NTD dimerizes through its C terminus, and like Atg17, the N terminus is located near the outer tips of the subunits in the dimer context. On the other hand, the FIP200 dimer gives rise to a C shape, in contrast to the S shape of Atg17 (Ragusa et al., 2012). The Atg17 S has been postulated to have a unique role in nucleating cup-shaped phagophores in bulk autophagy (Bahrami et al., 2017) on the basis of first-principles physical membrane modeling, something that would not be achieved by a C. Cup-shaped FIP200 structures have been visualized in amino acid–starved U2OS cells by super-resolution microscopy (Kenny et al., 2019 *Preprint*), likely corresponding to phagophores observed in cells by EM (Hayashi-Nishino et al., 2009; Ylä-Anttila et al., 2009). The ∼20-nm diameter of the FIP200 C shape is potentially compatible with localization on highly curved ATG9 vesicles or on the rim of the growing phagophore.

We mapped the mutual determinants for FIP200 and ATG13 binding to one another. Given that each of these proteins is central to autophagy, that they interact strongly with one another, and that the yeast Atg13-Atg17 interaction is functionally critical, we had expected that weakening this interaction would lead to a reduction in function. We confirmed that mutating the binding site for ATG13 on FIP200 and the FIP200-binding site of ATG13 impaired mitophagy, consistent with expectations.

The similarity in structure between the FIP200NTD and the combined ULD and scaffold domains of TBK1 (Shu et al., 2013; Zhang et al., 2019) was unexpected. Despite the limited resolution of the NTD cryo-EM structure, the positions of the helices and ULD relative to one another are unmistakably related and otherwise unique. This suggests to us that FIP200 is a structural composite of a TBK1-like N-terminal region and an Atg11-like C-terminal region. TBK1 in turn appears to be a structural chimera of a kinase with the FIP200NTD. Given increasing evidence for ubiquitous roles for TBK1 in mammalian autophagy initiation (Vargas et al., 2019), it should perhaps not be so surprising that the FIP200 and ULK1 system, collectively, and TBK1 have a structural, and presumably evolutionary, relationship.

We performed an intensive examination of the network of interactions between the ULK1 complex subunits and came to one more unexpected conclusion: that the complex contains a constitutive dimer of FIP200 but only one copy of every other subunit. The C-terminal end of FIP200NTD appears to be close to the center of the dimer interface; thus, the start of the dimeric coiled-coil domain projects away from this interface with matching symmetry. The division of labor of FIP200 domains seems to be that the Claw dimer at the very tip of the complex binds cargo, the coiled-coil acts as a long-range connector, and the NTD coordinates the ATG13:ATG101 subcomplex and ULK1 itself. As a stable dimer even at high dilution in our experiments, FIP200 seems unlikely to require cargo to dimerize. Therefore, a receptor-like model in which FIP200 transmitted a signal by undergoing cargo-induced dimerization seems unlikely to us. The 2:1 FIP200:ULK1 stoichiometry would prevent ULK1 from dimerization and auto-activating in the absence of higher-order clustering. This suggests an appealing mechanism for cargo-induced ULK1 activation in which multiple FIP200 dimers cluster on the autophagic substrate/cargo, bringing ULK1 monomers into proximity for autoactivation.

## Materials and methods

### Plasmid construction

The sequence of all DNAs encoding components of the ULK1 complex was codon optimized, synthesized, and then subcloned into the pCAG vector. All components were tagged with GST, MBP, or TwinStrep-Flag for affinity purification or pulldown assays. N-terminal GST, MBP, or TwinStrep-Flag tags may be followed by a tobacco etch virus cleavage site. All constructs were verified by DNA sequencing. Details are shown in Table S3.

### Protein expression and purification

All proteins used for NSEM, HDX-MS, and MALS analyses were expressed in HEK 293-GnT1 suspension cells by using the polyethylenimine (Polysciences) transfection system. Cells were transfected at a concentration of 2–2.5 × 10^6^/ml and harvested after 48 h. The harvested cells were pelleted at 500 *g* for 5 min at 4°C and then washed with PBS once. The pellets were then lysed with lysis buffer containing 50 mM Tris-HCl, pH 7.4, 200 mM NaCl, 2 mM MgCl2, 1 mM tris(2-carboxyethyl)phosphine (TCEP), 1% Triton X-100, 10% Glycerol, and protease inhibitors (Roche) before being cleared at 16,000 *g* for 30 min at 4°C. The supernatant was then incubated with Glutathione Sepharose 4B (GE Healthcare), Amylose resin (New England Biolabs), or Strep-Tactin Sepharose (IBA Lifesciences) as appropriate, with gentle shaking for 12 h at 4°C. The mixture was then loaded onto a gravity flow column, and the resin was washed extensively with wash buffer (50 mM Hepes, pH 8.0, 200 mM NaCl, 1 mM MgCl_2_, and 1 mM TCEP). The proteins were eluted with wash buffer containing 50 mM glutathione, 50 mM maltose, or 10 mM desthiobiotin as appropriate. In some cases, two affinity steps were used. Constructs containing Tobacco Etch Virus Protease (TEV) cleavage sites were treated with TEV at 4°C overnight. For HDX-MS and NSEM analyses, the protein was applied to a final SEC step before use. For the Strep-ATG13:Strep-ATG101 complex, a Superdex 200 column (GE Healthcare) was used, and for all other samples, a Superose 6 column (GE Healthcare) was used.

### Pulldown assays

10 ml of HEK 293-GnT1 suspension cells were transfected at the concentration of 2–2.5 × 10^6^/ml and harvested after 48 h. The harvested cells were pelleted at 500 *g* for 5 min at 4°C and then washed with 5 ml PBS once. The pellets were then lysed with 1 ml lysis buffer containing 50 mM Tris-HCl, pH 7.4, 200 mM NaCl, 2 mM MgCl_2_, 1 mM TCEP, 1% Triton X-100, 10% Glycerol, and protease inhibitors before being cleared at 12,000 rpm for 10 min at 4°C. The supernatant was then incubated with 20 µl Glutathione Sepharose 4B, Amylose resin, or Strep-Tactin Sepharose with gentle shaking for 8 h at 4°C. The protein-bound resin was washed with 1 ml lysis buffer three times and then eluted with 60 µl elution buffer containing 50 mM glutathione, 50 mM maltose, or 10 mM desthiobiotin, respectively. The eluted proteins were applied to SDS-PAGE for analysis. For Fig. 1 B, FIP200(N-640)–MBP and FIP200(636-C)–MBP were first purified by GST affinity purification, followed by TEV cleavage and then MBP affinity purification. The MBP-ULK1:Strep-ATG13:Strep-ATG101 subcomplex was purified by Strep affinity purification and then left on the resin. The resin was then mixed with FIP200 protein (final concentration: 200 nM) at 4°C with gentle shaking.

For Fig. 2 A, purified GST-FIP200, MBP-Strep-ULK1, and Strep-ATG13:Strep-ATG101 were used. The final buffer was 20 mM Hepes, pH 8.0, 200 mM NaCl, 1 mM TCEP, 5 mM desthiobiotin, and 1% Triton-X-100. The protein concentration was 200 nM. For Fig. 3 D, purified FIP200-MBP and GST-ATG13MR were used. The final buffer was 20 mM Hepes, pH 8.0, 200 mM NaCl, 1 mM TCEP, and 1% Triton-X-100. The protein concentration was 500 nM.

For quantification of the pulldown assay in Fig. 2 D and 3 D, ImageJ was invited to measure the intensity of bands in SDS-PAGE gel. The expression of GST-FIP200NTD was calculated as Intensity(GST-FIP200NTD in GST pulldown gel)/Intensity(Strep-ATG13 in Strep pulldown gel). Relative expression efficiency of GST-FIP200NTD was calculated as Expression(GST-FIP200 WT or Mutants)/Expression(GST-FIP200 WT). The pulldown of GST-FIP200NTD by Strep-ATG13:ATG101 was calculated as Intensity(GST-FIP200NTD in Strep pulldown gel)/Intensity(GST-FIP200NTD in GST pulldown gel)/Intensity(Strep-ATG13 in Strep pulldown gel). Relative pulldown efficiency of GST-FIP200NTD was calculated as Pulldown(GST-FIP200 WT or Mutants)/Pulldown(GST-FIP200 WT). The pulldown of GST-ATG13MR by FIP200NTD-MBP was calculated as Intensity(GST-ATG13MR in MBP pulldown gel)/Intensity(FIP200NTD-MBP in MBP pulldown gel)/Intensity(GST-ATG13MR in Input gel). Relative pulldown efficiency of GST-ATG13MR was calculated as Pulldown(GST-ATG13MR WT or Mutants)/Pulldown(GST-ATG13MR).

### HDX-MS

Protein samples for HDX were concentrated to a 10-µM stock before HDX. HDX was initiated by adding 95 µl of deuterated buffer containing 20 mM Hepes, pH 8.0, 200 mM NaCl, and 1 mM TCEP into 5 µl of protein stock at 30°C. Exchange was performed for 6 s, 60 s, 600 s, or 60,000 s and quenched at 0°C by the addition of 100 µl of ice-cold quench buffer (400 mM KH_2_PO_4_/H_3_PO_4_, pH 2.2). The 60,000-s sample served as the maximally labeled control. All HDX reactions were repeated three times. Quenched samples were injected into a chilled HPLC (Agilent) setup with in-line peptic digestion and desalting steps. The analytical column used was a Biobasic 8.5-µm KAPPA column (Thermo Fisher Scientific). The peptides were eluted with an acetonitrile gradient and electrosprayed into an Orbitrap Discovery mass spectrometer (Thermo Fisher Scientific) for analysis. To generate the gradient, solvent A was 0.05% TFA, while solvent B was 0.05% TFA in 90% acetonitrile. The elution method was as follows: 0–6 min: 10% B; 6–18 min: from 10% B to 55% B; 18–19 min: from 55% B to 90% B; 19–25 min: 90% B; 25–26 min: from 90% B to 10% B; and 26–30 min: 10% B. The spray voltage was set at 3.4 kV, capillary temperature was set at 275°C, capillary voltage was set at 37 V, and tube-lens was set at 120 V. As a control, unexchanged samples went through the same process, except that deuterated buffer was replaced by undeuterated buffer.

To identify peptides, unexchanged samples were analyzed by tandem mass spectrometry (MS/MS) analysis with the same HPLC method. Tandem MS/MS was performed using data-dependent analysis, in which a cycle of one full-scan MS spectrum (m/z 300–2,000) was acquired followed by MS/MS events (CID fragmentation). MS/MS was sequentially generated on the first to the 10 most intense ions selected from the full MS spectrum at a 35% normalized collision energy. The ion trap analyzer was used for MS2, activation time was 30 ms, and the dynamic exclusion was set at 30 s. For HDX mass analysis, only a full-scan MS spectrum was acquired, and the resolution was 30,000.

Database searches were performed with the Proteome Discoverer 2.1 (Thermo Fisher Scientific) using the Sequest HT search engine to identify peptides. Raw data were searched against the small database containing all four components of the ULK1 complex. The following search parameters were used: unspecific cleavage was used; precursor mass tolerance was set to ±10 ppm, and fragment mass tolerance was set to ±0.6 D. Target falase discovery rate was set to 1% as the filter cutoff for the identified peptides. For HDX analysis, mass analysis of the peptide centroids was performed using HDExaminer (Sierra Analytics), followed by manual verification for every peptide.

### NSEM collection and processing

Purified protein samples were diluted to ∼50–200 nM final concentration in running buffer immediately before application to glow discharged continuous carbon grids. Protein samples were stained twice with 2%–4% uranyl acetate and allowed to dry at room temperature. Samples were imaged using a T12 or F20 transmission electron microscope operating at 120 keV (Thermo Fisher Scientific) as indicated in Table S1. Data were manually collected and assessed for stain quality throughout data collection. F20 datasets used a recorded magnification of 89,000×, collected with an ultrascan camera at a pixel size of 1.5 Å/pixel (Gatan). T12 datasets were captured using a magnification of 49,000× with a 4k × 4k CCD camera (Gatan), which corresponds to 2.2 Å/pixel.

All datasets spanned a range of 1–4 µm defocus and a total dose of 30–50 e^−^/Å^2^. Single particles were selected using the Relion Autopicker (Zivanov et al., 2018) and extracted with the indicated box size (Table S1). Data were cleaned by 2D classification within Cryosparc2, removing classes that had no features or contained background picks. Final 2D classification into 80 or 50 classes was performed with the “uncertainty parameter” set to 8. This setting yielded the best distribution of class averages across all datasets.

### Size exclusion and MALS analysis

All light scattering experiments were performed with a running buffer of 50 mM Tris, 150 mM NaCl, and 1 mM TCEP, pH 7.8. Purified ULK complex sample was concentrated to ∼6–10 µM, and 100 µl was injected over a 24-ml Superose 6 Increase 10/300 GL column (GE Healthcare) in tandem with light scattering analysis using both an Optilab rEX differential refractive index and DAWN HELEOS II MALS detectors. Data were analyzed using ASTRA VI software (Wyatt Technology) with peak alignments, normalization, and band broadening effects determined from a standard of 2 mg/ml bovine serum albumin. The molecular weight of the sample was reported by selecting the peak with the largest UV intensity and averaging the molar mass values across the width of the peak. The radius of hydration was inconsistent across different runs and was not included in the analysis.

### Mitophagy assay

For CID assay, HeLa cells stably expressing mKeima-P2A-FRB-Fis1 and FKBP-GFP-ATG13 and mutants were treated with A/C heterodimerizer (for simplicity, it is called “rapalog” in the figure; Clontech; #635056) for 24 h and then subjected to FACS analysis as previously described (Vargas et al., 2019). For FIP200 KO rescue experiments, HeLa or FIP200 KO cells stably expressing mito-mKeima were cotransfected with 0.25 µg pEYFP-Parkin, 1 µg pHAGE-HA-FIP200-IRES-puro, and mutants with FuGENE HD (Promega) for 18 h and then treated with 10 µM Oligomycin (Calbiochem), 10 µM Antimycin A (Sigma), and 20 µM QVD (ApexBio) for 5 h before FACS analysis. All the constructs (including site mutagenesis) were made with Gibson assembly (NEB; #E2611S) and confirmed by Sanger sequencing. The complete sequence map of each construct is available upon request.

### Cryo-EM sample preparation and data collection

UltrAUfoil 2/2 300 mesh gold grids (Quantifoil) were used for open hole data collection (Data S1). Samples were concentrated after gel filtration to ∼5 μM and applied to glow discharged grids. Blotting was performed at 100% humidity in a Vitrobot Mark IV (Thermo Fisher Scientific) for 2–6 s. Graphene oxide–coated grid datasets were prepared as follows. UltrAUfoil 1.2/1.3 300 mesh gold grids (Quantifoil) were glow discharged under mild conditions (i.e., 10 mAmp for 15 s). Grids were incubated with a layer of polyethylenimine at 1 mg/ml for 2 min, after which the solution was blotted off using Whatman 1 filter paper followed by two rounds of washing with water. Grids were allowed to dry for 15 min and then incubated with 4 µl of graphene oxide flakes (Sigma) at ∼0.2 mg/ml for 1–2 min. Excess graphene oxide solution was wicked away and washed with two 4-µl drops of water. Coated grids were screened in a T12 microscope to assess coverage and quality of graphene oxide before plunge freezing. FIP200:ATG13MR samples were checked via NSEM directly before freezing to assess quality of protein and protein concentration. Protein samples for the graphene oxide datasets were diluted immediately before freezing in gel filtration buffer (50 mM Hepes, pH 8.0, 200 mM NaCl, 1 mM MgCl_2_, and 1 mM TCEP) to a final concentration between 200 and 500 nM. 3.0 µl of sample was loaded onto graphene oxide–coated gold grids. Sample was plunge-frozen using a Vitrobot Mark IV (Thermo Fisher Scientific) with blot force 10–20 for 3–7 s.

Data were collected by the same procedure for all datasets. Samples were clipped and loaded into a Talos Arctica (Thermo Fisher Scientific) operating at 200 kV. Frames were collected at 36,000× nominal magnification on a K3 direct electron detector (Gatan) in super-resolution counted mode at 0.5685 Å/pixel. Serial EM was used for automated image shift data collection of a five-target cross pattern. Movies were taken in 100-ms frames at ∼1 e^−^/frame, totaling an electron dose of 60 electrons per movie.

### Cryo-EM processing

The data processing scheme for the final maps is shown in Fig. S5. Micrographs were drift corrected using MotionCor2 (Zheng et al., 2017) and Fourier binned to 1.137Å/pixel. CTFFIND4 (Rohou and Grigorieff, 2015) was used to estimate the contrast transfer function parameters of the integrated micrographs. Micrographs were cleaned by inspection and fast Fourier transform quality.

The three datasets were collected and combined during processing as follows. For Datasets 1 and 2, particles were picked using the Relion Autopicker and extracted at a box size of 200 × 200 pixels with 2.274 Å/pixel. Dataset 1 was taken in open holes on a MBP-FIP200NTD:ATG13 (363–460) sample. Nearly half of the micrographs were removed due to poor drift correction, imaging of empty holes, or distorted CTF information. Relion Autopicker yielded ∼293k initial particle picks for this dataset. Single particles were pruned by 2D classification within Cryosparc2 (Punjani et al., 2017). Although extensive processing schemes were attempted, no high-quality 3D initial models were found. This we suspect is due to the shape of FIP200 and the low contrast of the protein in vitreous ice.

Dataset 2 was taken on graphene oxide with FIP200NTD: ATG13 (363–460) as the protein sample. Micrographs suffering from large graphene oxide creases were removed during early processing steps. Similar particle picking and pruning were performed on this dataset, and ∼200k initial particle picks were found. Combination of these datasets led to our final reconstruction via a soft mask around the highest density arm of the FIP200 dimer, as shown in Fig. S5 B. The final 29,198 particles were masked and processed using nonuniform refinement in Cryosparc2. Preferred orientation was seen throughout the steps of processing and was present in both orientation plot and 3DFSC (28671674) of the final single-arm map. The final reconstruction was locally filtered and sharpened with a b factor of −661 within Cryosparc2. Of note, multibody refinement, local motion correction, symmetry expansion, and additionally 3D classification did not improve the resolution of the final map. Processing within other software packages yielded maps of worse quality and higher degrees of anisotropic density.

Dataset 3 was taken to increase the initial particle count for the graphene oxide data. A similar number of micrographs containing graphene oxide creases was present and removed compared with Dataset 2. Dataset 3 was picked using a trained model within crYOLO (Wagner et al., 2019), which proved to center on the FIP200 dimer better than other particle pickers in our hands. 2D classification was used to prune Dataset 3 down to ∼80k final particles from an initial ∼159k particles (Fig. 6 A). Upon the realization that crYOLO was performing better for picking, Datasets 1 and 2 were reprocessed, leading to final particle counts of ∼65k and ∼69k particles, respectively. The final map was deposited into the Electron Microscopy Data Bank under accession code 21325.

Combination of the three datasets did not yield a better final resolution model, but it did reveal a large conformational landscape of the FIP200 dimer (Fig. 6 B). Conformations spanned a 60-Å range from ∼160 Å to 220 Å. This information helped to explain our unsuccessful attempts at multibody refinement and local symmetry expansion, as motion at this scale has not been resolved that we are aware of. Data were processed together in Cryosparc2 and pruned to a final heterogeneous classification of 213,156 particles. 3D classification into six classes yielded the final three maps, which show strong density for each arm of the FIP200 dimer. Similar to processing of the two dataset final maps, further processing did not yield higher resolution features.

For docking of the TBK1 and ATG17 structures into our final monomeric density map, we used the “Fit_in_Map” function within Chimera. For the TBK1 ULD/SLD structure, we removed the atomic coordinates of the Kinase domain before docking. The structure was first docked manually near the FIP200NTD monomer, and then 1,000 fits were sampled with a search radius of 2. The docking position with the highest cross correlation is shown in Fig. 6 D. Similarly, the ATG17 monomer was docked in the same manner, and the highest docked position is shown in Fig. S5 F.

### Online supplemental material

Fig. S1 shows raw micrographs and 2D class average galleries of all NSEM datasets. Fig. S2 contains deuterium uptake plots of significant peptides in the FIP200NTD and a quantitation of the pulldown assays in Fig. 2 and Fig. 3. Fig. S3 contains the raw FACS plots, which are quantitated in Fig. 2 G and Fig. 3 E. Fig. S4 shows the SDS-PAGE pulldown assay of the ULK1 complex in the presence of ATG13-ΔC. Fig. S5 contains a workflow of the cryo-EM data along with a sequence alignment between FIP200 and TBK1. Table S1 contains metrics for each NSEM dataset. Table S2 shows processing and data collection metrics for the FIP200NTD cryo-EM datasets. Table S3 lists all constructs used in this study. Data S1 contains summary information on the HDX-MS datasets.

## Supplementary Material

Table S1 contains metrics for each NSEM dataset.

Table S2 shows processing and data collection metrics for the FIP200NTD cryo EM datasets.

Table S3 lists all constructs used in this study.

Data S1 contains summary information on the HDX-MS datasets.
